# Nutritional Aspects in Diagnosis and Management of Food Hypersensitivity—The Dietitians Role

**DOI:** 10.1155/2012/269376

**Published:** 2012-10-24

**Authors:** Carina Venter, Kirsi Laitinen, Berber Vlieg-Boerstra

**Affiliations:** ^1^The David Hide Asthma and Allergy Centre, Isle of Wight PO30 5TG, UK; ^2^University of Portsmouth, 2 King Richard 1st Road, Portsmouth PO1 2FR, UK; ^3^Functional Foods Forum and Institute of Biomedicine, University of Turku, 20014 Turku, Finland; ^4^Department of Pediatric Respiratory Medicine and Allergy, Emma Children's Hospital, Academic Medical Center, University of Amsterdam, Meibergdreef 9, 1105AZ Amsterdam, The Netherlands

## Abstract

Many common foods including cow's milk, hen's egg, soya, peanut, tree nuts, fish, shellfish, and wheat may cause food allergies. The prevalence of these immune-mediated adverse reactions to foods ranges from 0.5% to 9% in different populations. In simple terms, the cornerstone of managing food allergy is to avoid consumption of foods causing symptoms and to replace them with nutritionally equivalent foods. If poorly managed, food allergy impairs quality of life more than necessary, affects normal growth in children, and causes an additional economic burden to society. Delay in diagnosis may be a further incremental factor. Thus, an increased awareness of the appropriate procedures for both diagnosis and management is of importance. This paper sets out to present principles for taking an allergy-focused diet history as part of the diagnostic work-up of food allergy. A short overview of guidelines and principles for dietary management of food allergy is discussed focusing on the nutritional management of food allergies and the particular role of the dietitian in this process.

## 1. Introduction

According to the World Allergy Organisation [1] about 1.9%–4.9% of children suffer from cow's milk allergy, with the prevalence in adults much lower; less than 0.5% [2]. It is known that perceived food allergy could be 10 times higher [3, 4] than that confirmed by appropriate tests. Although a large number of foods are suspected to cause food allergies, most studies have focused on 6 common foods which include cow's milk, hen's egg, soya, peanut/tree nuts, fish/shellfish, and wheat, clearly showing different patterns based on the population studied and the diagnostic methods used [5]. Peanut allergy is one of the most common causes of food-induced anaphylaxis, with a reported or challenge proven prevalence rate between 0.06% and 5.9% depending on the country and age group studied [2]. Tree nut allergies also show a wide range of reported or challenge-confirmed prevalence ranging between 0.2%–8.6% [2]. High rates of reported allergies to tree nuts may, however, be due to cross-sensitisation with aeroallergens, rather than a primary food allergy, with an increased number of patients with so-called oral allergy syndrome/fruit pollen syndrome seen [6].

There is a paucity of information on the role of nutrition versus just food avoidance in the management and natural course of food allergy. Very little is also known about the effect of a nutrition consultation in this process. Furthermore, the role of the dietitian and the diagnostic and therapeutic value of the elimination diet has not been established and extensively investigated. A systematic review on the role of the dietitian and the value of the elimination diet in the diagnostic and therapeutic phase found only two papers. These only partially address the issue and focus mainly on the nutritional status of children with food allergies [7, 8]. The lack of information on how to take an allergy-focused diet history, linking symptoms of allergic disease with foods implicated in the adverse reactions, and the impact of general nutritional intake, has been recognised by the European Academy of Allergy. A special task force initiated by the Interest Group on Allied Health is addressing this.

The paucity of information on the role of nutrition in allergy is reflected in and may even be explained by the fact that globally seen, only a few dietitians and nutritionists have both a specialty in food allergy and are academically trained in food allergy. The level of training in dietetics in general differs between countries and the UK is the only country which offers MSc degrees in allergy (http://www.southampton.ac.uk/ and http://www.imperial.ac.uk/). As a result, the level of knowledge about food allergy not only differs strongly between individual dietitians but also strongly between countries. A survey conducted in the USA [9] and UK [10] shows a great need for food allergy knowledge amongst dietitians. Dietitians in the USA felt more knowledgeable about the definitions of food allergies (57% scored “high level of knowledge” in the USA versus 31% in the UK, *P* < 0.001) and intolerances (59% scored “high level of knowledge” in the USA versus 30% in the UK, *P* < 0.001). However, UK-based dietitians indicated more confidence in designing food challenge protocols (12% UK versus 8% USA scored “high”, *P* = 0.12) and 18% in the UK and 19% in the USA indicated to have no proficiency in developing challenge protocols. Very interestingly, dietitians from both countries indicated that their most immediate need was standardised patient handouts/diet sheets (89% USA and 70% UK).

Having realised this problem some time ago, the International Network for Diet and Nutrition in Allergy (INDANA) (http://www.indana-allergynetwork.org/) was established. The aim of INDANA is to encourage, support, and disseminate best evidence-based clinical practice and to influence the development of the dietitian's and nutritionist's role in the field of food hypersensitivity (FHS) at international level. In this paper, the aims and activities of INDANA will be further discussed.

The role of the dietitian differs between countries depending on the extent of the physician's role in the dietary management. In some countries the dietitians are trained to be involved in the diagnosis, while in other countries the dietitians are involved in the dietary management only. 

During 2010 and 2011, three official guidelines have been published on the diagnosis and management of food allergies by international organizations or by comprehensive working groups. These are the World Allergy Organisation guidelines on the diagnosis and management of cow's milk allergy (DRACMA guidelines) [1], the USA guidelines on the diagnosis and management of food allergies in adults and children [11] and the UK NICE guidelines on the diagnosis of food allergies in children [12]. 

All 3 guidelines recognize the important role of nutrition education. However, the UK NICE guidelines were the only guidelines that recognized the role of the dietitian in diagnosis and management of food allergies [12]. The DRACMA guidelines mention the role of the dietitian (referred to as a nutritionist) in the management of cow's milk allergy [1]. 

## 2. Aim

This paper sets out to present nutritional aspects in diagnosis and management of food allergies, comprising

- the aims and activities of the International Network for Diet and Nutrition in Allergy (INDANA);
- principles for taking an allergy-focused diet history in the diagnosis of food allergy;
- principles for the nutritional and dietary management of food allergy, including the impact of food allergy on an individuals diet and nutritional status, unnecessary avoidance diets and dietary restrictions, and psychosociological aspects of food allergy.

## 3. International Network for Diet and Nutrition in Allergy: INDANA

Acknowledging the need for education on food hypersensitivities for both dietitians and other professionals, the International Network for Diet and Nutrition in Allergy (INDANA) was established in 2009 by a group of academic dietitians and food scientists specializing in food allergies and intolerances. This nonprofit international network [13] aims to

- develop the role of the dietitian/nutritionist in the field of food allergy and to enhance the focus on diet and nutrition when dealing with food allergy;
- provide a platform for nutritional and dietary management advice for all those involved in dietary care of food allergy;
- educate health care professionals (HCPs) involved in food allergy on nutrition and dietary management of food allergy;
- encourage international collaboration and research;
- encourage a membership that is representative of all countries and continents where food allergy is highly prevalent.

INDANA aims to bridge the gap in science between food hypersensitivity, immunology, nutrition, and food science to improve the prevention, nutritional diagnosis, and management of those living with food allergies and intolerances. 

The steering group consists of members with a first degree in Nutrition & Dietetics or Nutrition, plus postgraduate training in allergy or a research degree at Masters or Doctorate level. The steering committee includes representation from the USA, Europe, Australia, and South Africa. The three authors of this paper are members of the steering committee of INDANA. Membership is open to any HCP with a relevant first degree who is working in the field of food hypersensitivity (i.e. dietitian, nutritionist, nurse, clinician, researcher, and industry). 

The importance of diet and nutrition in allergic disease and the role of the dietitian/nutritionist are now officially acknowledged by the European Academy of Allergy and Clinical Immunology (EAACI), by initiating an Interest Group on Diet and Nutrition in the Allied Health (IG on AH). This creates a platform within the EAACI where people, sharing an interest and expertise in diet and nutrition in allergy, can meet, teach, exchange expertise and ideas, and support the EAACI with regard to this topic. Also recently INDANA was officially incorporated in the American Academy of Allergy, Asthma and Immunology (AAAAI).

The primary goals of INDANA and the IG on AH are to educate HCPs on food hypersensitivity, taking into account international variations in the role of the dietitian in the diagnosis and management of these conditions. The IG on AH is involved in setting up an EAACI-initiated practical training course on food allergy and a Food Allergy and Anaphylaxis Guideline. In addition, members of INDANA and the IG on AH are also presenting at international conferences of the EAACI, AAAAI and the International Congress of Dietetics (ICDs). The first educational and research tool that is being developed is a standardized allergy-focused diet history. This tool is being developed under the funding of the EAACI by the Interest Group (IG) on Allied Health (AH). This tool could be adjusted for use in the USA pending support of national allergy organisations. This may be particularly useful for physicians who do not have access to dietitians. 

The next steps will be to develop educational materials for health care professionals around the globe and to initiate research projects.

## 4. Principles for Taking an Allergy-Focused Diet History 

The aim of the allergy-focused diet history is to investigate if there is an association between food or diet and the symptoms of the patient.

When patients have immediate food allergic reactions and reactions to single foods, it is often obvious to which food the patient has reacted. However, very often patients have chronic symptoms, reactions to compound foods, or sensitisation to foods of which the clinical relevance is not clear. This may be due to unknown allergens, may be caused by cross-sensitisation between pollens and foods [14], or due to a deficient or unbalanced diet. The outcome of the allergy-focused diet history may direct the physician to further diagnostic testing supporting the final diagnosis by the physician and may direct the dietary measures to be taken. A detailed allergy-focused diet history should consist of the following.

### 4.1. Assessment of Signs and Symptoms and Possible Mechanisms

According to the UK NICE guidelines, the possibility of food allergy should be considered in children and young people who are presenting with one or more allergic signs and symptoms, in particular when there are persistent symptoms that involve different organ systems [12]. These symptoms may be IgE-mediated or non-IgE-mediated or may not involve the immune system. 

### 4.2. Allergy-Focused Clinical History and Linking Allergenic Foods to Symptoms

If food allergy is suspected (by a HCP parent, carer, child, or young person), a HCP with the appropriate competencies should take an allergy-focused clinical history tailored to the presenting symptoms and age of the child, young person, or adult.

This should include [12] information on the following:

- any personal history of atopic disease (asthma, eczema, or allergic rhinitis);
- any individual and family history of atopic disease (such as asthma, eczema, or allergic rhinitis) or food allergy in parents or siblings;
- details of any foods that are avoided and the reasons why;
- an assessment of presenting symptoms and other symptoms that may be associated with food allergy, including questions about:
  - the age of the child or young person when symptoms first started;
  - speed of onset of symptoms following food contact;
  - duration of symptoms;
  - severity of reaction;
  - frequency of occurrence;
  - setting of reaction (for example, at school or home);
  - reproducibility of symptoms on repeated exposure;
  - what food and how much exposure to it causes a reaction;
  - cultural and religious factors that affect the foods they eat;
  - who has raised the concern and suspects the food allergy;
  - what the suspected allergen is;
  - the child or young person's feeding history, including, the age at which they were weaned and whether they were breastfed or formula-fed, if the child is currently being breastfed, considering the mother's diet;
  - details of any previous treatment, including medication, for the presenting symptoms and the response to this;
  - any response to the elimination and reintroduction of foods (NICE).

**Additional Information on Consumption of the Major Allergenic Foods, Food Chemicals, and Any Possible Cross-Reactions**

- Do you regularly eat the following foods (peanuts, tree nuts, sesame seeds, celery, milk, egg, wheat, fish, shell fish, molluscs, soya, lupin, mustard, or sulphite containing foods) and do you experience any problems when eating them?
- Do you have hay fever or are you allergic to pollens?
- It is important that the person taking the allergy-focused diet history has knowledge of all aspects of food allergy. OAS or oral itch is a type of food allergy classified by a cluster of allergic reactions in the mouth in response to eating certain (usually fresh) fruits, nuts, and vegetables [15]. However, within the spectrum of OAS lies the pollen-fruit syndrome (PFS), characterised by much milder symptoms and needs much less stringent dietary restrictions. It is now widely reported that OAS is becoming a widespread problem in Europe, particularly in younger age groups [16]. In the UK, the problem in the adult population is growing [17], but the number of children suffering from this problem is unclear. Information about OAS is particularly important for successful management of fruit, vegetable, and nut allergies in order to prevent unnecessary restrictions in these people's diets. PFS is not known to cause anaphylaxis but true IgE-mediated allergies to fruit, vegetables, and nuts, which are on the other end of the OAS spectrum can be potentially fatal, and it is important to distinguish between the two.
- Do you eat mainly home-cooked or commercially prepared foods? This will enable and prompt further questioning about possible chemicals in food.

**Factors to Take into Account during the Decision Making**

- What is the clinical relevance of positive test results if tests have been done?
- Is there any cross-sensitisation between foods and aeroallergens?
- Are there any extrinsic factors involved: medication, stress, exercise, alcohol, hormonal, infection, vitamin and mineral supplements and herbal medication.

### 4.3. A General Diet History to Assess the Quality of the Diet

Chronic symptoms may also be caused by other nonallergenic factors or may be caused by an unbalanced or deficient diet with similar presentation to food intolerance. Ask general questions focusing on nutrients of importance, particularly if a dietitian is in post. This can be done by dietitians using 2–7 days food diaries, a 24 hours recall diet history, or a more general diet history [18, 19]. The allergy specialist dietitian could be the ideal HCP to deal with this part of the diet history, having knowledge on food allergy, nutrition, and foods.

**When to Refer to a Dietitian**

- The more foods avoided the more important it is to refer to the dietitian [7];
- general growth monitoring and nutritional assessment, particularly children with growth issues either faltering growth or malnutrition [8];
- for help and advice on infant formula/milk substitutes and weaning [1];
- for advice on the general nutritional aspects of the diet and for psychological support [20];
- for information on life-style issues when living with food allergies such as, eating out, travelling, school trips/camps [21].

## 5. Principles for Management of Food Allergy and Intolerance 

The key treatment of food allergy is avoidance of the allergenic foods. Although this sounds very simple, dietary management encompasses more than advice on avoidance of the allergenic foods. Scientific data are scarce, but there is general agreement that the aims and principles of the dietary management of food allergy are multiple and include the following:

1. obtaining relief of symptoms by avoidance of the allergenic foods;
2. preventing inadvertent exposure to the allergenic foods;
3. preventing the patient from unnecessary avoidance of foods;
4. supporting normal growth and development for age and gender in children;
5. providing an adequate, healthy, nutritionally dense, and balanced diet with appropriate alternatives for the excluded food allergens to minimize the impact on quality of life [22].

The dietary management of food allergies can be complex and difficult to follow in most cases and input from a dietitian is very important. For optimal dietary management it is very important to know which foods should be avoided in order to give appropriate avoidance advice [20]. Without clear identification of the allergenic foods dietary management of food allergy becomes difficult. Additionally, a clear diagnosis enhances appropriate coping strategies and determines the level/degree of avoidance required.

In general, stringent dietary advice may be needed for food-allergic patients, including the avoidance of the following [23]:

- foods containing allergenic ingredients, even in small amounts;
- foods having a high risk for cross-contamination, such as chocolate in the case of peanut or cow's milk allergy/foods with precautionary labeling, although an individual risk assessment is recommended by some clinicians;
- unrefined oil derived from allergenic foods;
- meals or foods of uncertain composition, (for example, away from home).

Regulatory legislation is very helpful when allergenic foods are to be avoided as these allergenic foods must be declared on the label of prepacked foods. For the EU these are milk (including lactose), egg, soy, wheat or gluten, peanut, tree nuts, sesame, fish, crustaceans, molluscs, celery, lupin, mustard, and sulphites [24]. However, dietary management becomes much more complex when other foods leading to severe reactions have to be avoided. 

The dietitian should educate the patient about this legislation, about reading labels, about high-risk foods, and high-risk settings such as eating out, to be stringent if medical care is not nearby and about early signs of severe reactions. Advice for school meals, day care, birthday parties, holidays, and other social circumstances should also be addressed. All of these points should be included and discussed in an adequate dietary management plan [23].

Another aspect of food hypersensitivity is that many patients choose to avoid too many foods and therefore overrestrict their diets [3, 25]. Patients may avoid foods related to the allergenic food, for example, peanut allergic patients may avoid all other nuts and sesame, while this is not always indicated. Anxiety of unfamiliar foods and fear of inadvertent allergic reactions may also lead to unnecessary avoidance.

Anecdotal evidence indicates that food allergic patients live with a permanent alertness as to what they are eating in numerous situations and settings. Successful avoidance of foods requires an understanding of label reading, meal preparation, and effective communication to friends and restaurant personnel providing food [20, 26]. The knowledge of the potential dangers of an accidental exposure may lead to a heightened level of anxiety [27] and negatively impacts the quality of life [28], as well as affecting lifestyle issues and welfare [29]. Previous research studies have shown that food allergic consumers experience difficulties when eating out and while shopping [30, 31].

The number of studies looking at health-related quality of life (HRQoL) in those suffering from food allergies has increased over the years. These studies showed that HRQoL in patients with food allergy and their families was significantly reduced [29, 32–35]. They found that several areas of HRQoL are affected, such as, family and social activities, emotional issues, and family economy, basically all aspects of family life. Food-hypersensitive children are to a large extent also limited in performing social activities without adult supervision.

Patients should therefore be encouraged to expand their diet using foods which have been proven to be safe in a graded fashion. Specific ready-to-use introduction schedules for use at home for first exposure to foods have been developed and published and could also be used for reintroduction of foods [36]. 

Several patient groups deserve specific attention in dietary management, such as, lactating women and young children who need weaning/introduction of solids advice and advice on the most appropriate choice of hypoallergenic formula, picky/faddy eaters, and vegetarians. 

It is recommended that lactating mothers with food allergic children should remove the offending food from their own diet for a period of time.

These foods can be reintroduced once the infant/child improves in order to establish whether they (the mother) need to continue with the food avoidance [1, 12, 37]. Mothers avoiding cow's milk from their diet should be supplemented with calcium and vitamin D according to the national guidelines for each country [38]. In most countries, vitamin D supplementation is suggested for all breastfeeding women, irrespective of avoiding cow's milk or not.

Choosing the right formula for the patient based on the clinical presentation is a matter of huge debate with clear differences between countries. This choice should ultimately be based on clinical presentation, nutritional composition of the formula, residual allergenicity of the formula, and other components added to the formula. The DRACMA guidelines [1] performed a comprehensive review of the literature and their different indications for formulas suggest the use of an aminoacid-based formula for anaphylaxis, Heiner syndrome, and esinophilic Eosophagitis, with the use of extensively hydrolysed formula for all other clinical presentations. Three additional papers, however, suggest the use of aminoacid-based formulas for growth faltering, severe atopic dermatitis, multiple food allergies, and infants not responding to maternal avoidance of cow's milk [37, 39, 40]. One should, however, always take national differences into account as some countries may use soya formula as a first-line approach for some clinical conditions in infants over six months [41].

Weaning is a particular problem in infants suffering from food allergies, and the most difficult question is when and how to introduce other highly allergenic foods into the diets of infants with milk/egg allergies with parents understandably being cautious about introducing these foods [42]. For both nutritional and developmental reasons [43], over restriction and delayed introduction of these foods are not recommended. Another clinical dilemma is whether to “screen” for other food allergies in children with one food allergy. There are difficulties with interpreting the results of these tests in young infants which may lead to over restriction of foods to which an infant is sensitised but not allergic. The National Institute of Allergy and Infectious Diseases (NIAID (USA)) guidelines [11] states that there is insufficient evidence to suggest whether, or which, foods should be tested prior to introduction in children at risk of food allergies (either from a high-risk family or with other existing food allergies). Testing prior to introduction could potentially prevent allergic reactions, but there is currently no practical consensus on which (if any) foods should be tested.

Until further clear evidence becomes available, each clinician may have their own preference in dealing with the problem, but it makes sense to start with low-allergenic foods first, try the cooked version of a food first, increase the amount given, and expand the diet as soon as possible. Parents should be clear on how to deal with any unexpected reactions and realise that children with coexisting asthma/wheeze are more likely to have (perhaps) severe reactions [44].

Food allergic individuals should also be followed up at regular intervals. New allergens can emerge or patients can outgrow one or more of their food allergies. It should be obvious that in multiple and complex food allergies the management of food allergy is tailored to the individual patient and requires sophisticated skills and knowledge about food allergy and composition of foods from the dietitian. However, food allergy specialist dietitians are limited. Further education activities should be undertaken to educate more dietitians in diet history taking and the management of food allergy. It is one of the aims of INDANA to enhance the food allergy skills of dietitians around the world. 

## 6. Impact of Allergy on Growth of the Children 

Dietary antigens induce a local hypersensitivity reaction impairing the intestine's barrier function, leading to continuation of inflammation. The consequences of the inflammatory responses may be severe and manifested as impaired growth, increased symptoms, and poor quality of life. The cornerstone of the management of documented food allergies is an elimination diet and when appropriately designed and accomplished it dampens the inflammation and ensures optimal growth and well-being of the child. Nutritional inadequacies in food allergic individuals may result particularly from the elimination of multiple foods or nutritionally key foods, such as, milk or cereals [7, 8]. Early onset of symptoms, manifested during the first few months of life, compared to late onset, 6 to 10 months of age, appears to result in more seriously affected disease and the delay in growth may be more pronounced [45]. A Brazilian study [46] demonstrated the impact of food allergy on a child's nutritional status as the prevalence of poor growth was seen in as many as a quarter of children diagnosed with cow's milk allergy at the age of 24 months or less. A weight-for-age Z-score below 2 was demonstrated in 15.1% and a height-for-age Z-score below 2 in 23.9% of the children. Importantly, delay in diagnosis and thus delay in initiation of an appropriate dietary management may slow down growth. Conversely early diagnosis is associated with an appropriate growth for age, along with shorter duration of symptoms, fewer food allergies, and improved prognosis. 

There are a number of factors that could lead to poor growth in children with food allergy as summarised in Table 1. Nevertheless, severe cases of growth failure are rare, affecting only a minor proportion of children following an appropriate care plan and may relate to the severity of the disease, like a large skin surface area affected in atopic eczema [47]. Lack of appropriate care or poor compliance may result in severe nutritional inadequacies, for example, where small children have been fed with rice beverages with an inappropriate nutritional composition for the needs of a small child [48]. There is little evidence on the impact of food allergy on a child's body composition or bone health, but these may be potentially affected.

The mechanisms for impaired growth may rise from a sustained inflammation and subsequent reduced bioavailability or loss of nutrients in the gastrointestinal tract [49], while metabolic requirements may be increased. Patients may consciously or unconsciously regulate symptoms of the disease by (unnecessary) elimination of foods [50]. A limited diet may also be of psychosocial origin, particularly if food allergy is potentially life threatening as in some cases of peanut allergy [51]. Patients may also develop food aversions and anxiety, resulting in inadequate dietary intake or replacement of allergenic foods by foods that are not nutritionally equivalent. Some parents seek help for their child's symptoms from alternative practitioners, and unfortunately they may prescribe very limited diets with subsequent impact on dietary variety and nutritional status [42]. In this case, appropriate allergy testing and reintroduction of foods to the child's diet under dietetic supervision resulted in regain in weight and general improvement of health. 

Differences in food and nutrient intakes between children with and without food allergy have been reported. Dietary intake studies in children with food allergy compared to figures for healthy children or reference nutrient intakes have demonstrated lower intakes of energy and protein as well as that of zinc, iron, calcium, vitamin D, and vitamin E [52]. Deviations in growth may reflect a lower dietary intake of energy and nutrients or relate to allergy itself through inflammation. Indeed, a recent study showed that despite a deviant growth between healthy children and those with food allergy, no difference in dietary intake was detected [7]. This study again points to the need for nutritional evaluation of children with food allergies, preferably by allergy specialist dietitians. 

Poor growth does not seem to be a feature of allergy per se but rather a matter of inadequate dietary intake with reference to the requirements. Faltering growth may culminate in patients not being regularly monitored by HCPs specializing in allergy, including a dietitian, as the reasons for growth failure are various. Prospective follow-up studies have demonstrated that with careful monitoring of growth and management of allergy in early childhood, the growth of children remains within population reference values and only a minor proportion evince poor growth [53]. In addition, an early diagnosis of food allergy and subsequent initiation of dietary management enable catch up growth and normal adult height [54]. Nevertheless, nutritional risks persist as a study reported that at the age of 7 to 15 years lower height and weight Z-scores were detected in children who had avoided two or more foods, particularly milk, at the age of three years due to allergic symptoms [55]. This is indeed demonstrated by a range of studies, including previously presented cases [51] as well as the case study of a boy with multiple food allergies presented in the next chapter. 

Case 3 presents a boy with severe eczema associated with multiple food allergies. 

## 7. Conclusions 

An early diagnosis and appropriate care of food allergy are necessary to allow a good quality of life and nutritional status of the patient. An allergy-focused diet history assisted by the allergy-specialist dietitian may direct the physician to further diagnostic testing; supporting the final diagnosis dietetic expertise is important to conduct a dietary assessment to ensure appropriate intake of energy and essential nutrients and to provide patient-oriented counselling. This includes education on reading labels, safe eating at restaurants, risks of cross-contamination, and potential sources of hidden allergens but also informing about support groups and online resources. These patient-oriented tools facilitate the appropriate management and followup of patients with food allergies. Nonprofit organisations, such as the recently founded INDANA, focus on increasing awareness, education, and improvement of nutritional and dietary care in food allergy.

## 8. Case Studies

**Case 1 (a teenage girl with anaphylaxis to peanut)**

Lisa was referred to the allergy specialist dietitian by the paediatrician because of episodes of abdominal pain and cramps with increasing severity and several food allergic reactions over the years. The family attributed these reactions to peanut, because she was diagnosed with peanut allergy since she was young. However, they had been avoiding peanut strictly.

The paediatrician recently updated the information on sensitization to foods and inhalant allergens. Sensitization to inhalant allergens was negative, sensitization to foods was positive for the following foods: hazelnut 0.61 kU/L, peanut 76.2 kU/L, pistachio 7.14 kU/L, soy 2.72 kU/L. 

Sensitisation to coconut, almond, cashew nut, milk, egg, fish, and wheat was negative.

*Clinical and Allergy-Focused Diet History. *There is a positive family history of atopy and asthma in her father's family. The clinical history revealed that she has suffered from abdominal pain since she was very young. She reported increasing episodes of having cramps and nausea. She has asthma which was under good control. She was carrying an epipen for her peanut allergy, however, was not confident about using it.

Firstly, the diet history focused on the clinical relevance of the foods sensitized to and on excluding dietary errors on peanut ingestion.

Lisa has been trying to avoid peanuts and other nuts since she was young. The family read labels and try to avoid foods containing peanut and nuts. Foods with advisory labeling for peanuts and nuts were avoided.

Over the years, several allergic reactions occurred after eating a food or meal, of which the family thought that peanut must have been the causative ingredient. The reactions did not occur immediately. Remarkably, most reactions, except the reaction to M&Ms, were preceded by a form of exercise after eating (gym, playing outside, karate lessons) or stress (school party).

- When she was much younger she reacted to M&Ms with vomiting.
- When she was 11 years old, she had an anaphylactic reaction following a home made Indonesian meal without nuts or peanut, for which she used her epipen. The meal included tofu and soy sauce.
- Last year, at a school party, she reacted with swollen lips, itchy ears, and dyspnoea after eating a chicken nugget.
- A few months ago she reacted with swollen lips, tachycardia, and presyncope after eating meat coated with bread crumbs.
- Over the years there were several reactions of tachy- cardia, abdominal pain with cramps, and lip swelling having had commercially prepared meat products.
- On one occasion she had a hazelnut and a cookie with almonds without symptoms.

Secondly, the diet history focused on the nutritional composition of the food to rule out any over or under consumption of foods, because cramps and nausea may be related to fibre consumption. Lisa's diet, however, seems to be sound with no nutritional imbalances.

The dietitian also suspected the sensitisation to soy to be of clinical relevance and suspected exercise-induced anaphylaxis to soy. The paediatrician agreed with this, and the dietitian advised Lisa and her family to avoid not only peanuts and nuts, but also soy protein. Soy protein was incorporated in the Indonesian meal and could have been incorporated in the chicken nuggets, coated meat with bread crumbs, and commercially prepared meals.

*Results*. No anaphylactic reactions occurred from that point forward, and the complaints of abdominal pain, cramps, and nausea disappeared. The dietitian advised to continue avoiding peanuts, nuts, and soy protein and to take particular care when exercising. The avoidance of all other nuts needed further consideration. The paediatrician/allergy nurse updated the information and the use of the adrenaline autoinjector.

*Teaching Points*

- An allergy specialist dietitian can sustain the diagnosis in taking an allergy-focused diet history.
- The diet history is the cornerstone of the diagnosis of food allergy and may direct the nature of the foods to be avoided.
- The dietary history may reveal if chronic symptoms may be caused by a deficient or unbalanced diet and if external factors may play a role, such as, exercise.

*Reason for Referral to an Allergy Specialist Dietitian.* In many countries, access to the dietitian is limited, and allergy specialist dietitians are scarce. 

Referral is specifically important:

- when an allergy-focused diet history is required to examine if certain foods provoke symptoms or, in case of chronic symptoms, these complaints may be caused by a deficiency or imbalance in the diet;
- when nutritional adequacy of the diet is questionable and needs to be checked, for example, in case of avoidance diets in young infants and toddlers (specifically cow's milk free and wheat free diets), multiple food allergy, picky eaters, faltering growth, and when symptoms do not improve despite adequate medication;
- when counselling and nutritional management are required, for example, in patients having questions about the practical implication of the avoidance diet (replacements of foods, social activities, school meals, school camps, holidays), allergic reactions despite following an avoidance diet, anxiety to food, and overrestriction of foods.

**Case 8 (11 months old boy with multiple food allergies)**

 

*Referral *

- Oscar was referred to the allergy specialist dietitian by the paediatrician because of acute urticaria and angioedema after ingestion of egg and growth faltering. Skin prick test results indicated;
- egg allergy (8 mm SPT);
- peanut sensitisation (SPT 4 mm);
- no other positive SPT/IgE for milk, wheat, fish, soy.

*Clinical History.* There is a positive family history for atopy and asthma in both families. The clinical history reveals that he has suffered from eczema from about 2-3 months. He also has “loose” stools but does not wheeze.

*Allergy-Focused Diet History*: *Enquire about Breastfeeding, Formula Feeding and Weaning onto Solids, and If Any Reactions Occurred. *Oscar was breastfed until 6 months and received a top-up drink of cow's milk formula from 1 month of age. Solids were introduced at 5 months starting with baby rice, vegetables, and fruit. At six months gluten was introduced then chicken, lamb, fish, and lentils. No reactions were noticed to these.

*Enquire about the Introduction of the Major Allergenic Foods*

 Milk given from 1 month and mother did not avoid milk during lactation with no noticeable reactions.
 Wheat was introduced at 6 months with no noticeable reactions.
 Fish was given at about 7 months with no noticeable reactions.
 Egg was given at 10 months when he had the reaction.

He has not had peanut, tree nuts, sesame seeds, shell fish, molluscs, mustard, celery (although she thinks it might have been in a stew she made), lupin (although difficult in the UK to know which foods contain these), soya (not sure but as she cooks everything at home it seems to be unlikely).

*General Diet History.* Mother offers 3 meals per day which are nutritionally balanced, but he has always been a “difficult feeder”. He has breakfast most days (baby rice with apple) but often refuses lunch (usually have 1*⁄*2 banana) and needs distraction most meal times but may eat about 90 g of dinner with pasta/rice/potato, meat, and mixed vegetables.

He has 350 mL/day of formula, which he drinks in 5-6 bottles of 60 mL/bottle.

*Dietary Assessment.* There are some concerns about his intake of protein and energy (60% of required calculated intake), iron (75% of UK RNI), calcium (51% of UK RNI), and vitamin D (60% of UK RNI).

**Dietary Management**

 

**Initial Dietary Intervention**

The initial dietary consultation included advice on an egg and nut-free diet. It was felt that the diagnosis of egg allergy was clear. The peanut result clearly only indicated sensitisation at this stage rather than clinical allergy as he has not consumed peanut until now, but it was felt that he was too young for a peanut challenge.

The formula was changed to an energy dense formula of 100 kcal/100 mL and 2.5 g protein/100 mL. A normal infant formula contains approx 67–79 kcal/mL and 1.4–1.7 g of protein. Mother was also advised to further increase his protein, kcal, and calcium intake with cheese and to add double cream to sauces. Meal times should be kept to 30 min and a long consultation about dealing with faddy eating, including, practical tips follows. No force feeding was allowed and mother was asked to allow time for messy play. Inclusion of red meat (iron) and fatty fish (vitamin D) when possible was recommended, but these deficiencies should be corrected by taking sufficient formula, to initially aim to increase formula to 500 mL per day, which would not provide all the nutrients he needs but a step in the right direction.

**After Dietary Intervention**

After the dietary intervention, his volume of feed was reduced and he was only managing 30 mLs per bottle that is about 180 mL per day. His “difficult feeding behaviour” turned into extreme food refusal, and he was crying during and after feeds showing clear signs of discomfort. His eczema also seemed to flare after every meal. He stopped gaining weight.

**Results: Followup and Current Intervention**

He started on a cows' milk and soya-free diet and changed formula to amino-acid-based formula. The decision about soya avoidance was based on the concomitant soya allergy seen in children with gut symptoms and a non-IgE-mediated (in this case) milk allergy. His eczema cleared completely, feeding standard improved, and he started to gain weight again. The patient is currently on egg- and nut-free diet with plans to review the nut-free diet and possible challenge after 12 months. Both the suspected milk and soya allergies also need to be confirmed by challenge, but there are currently no standardized procedures for performing food challenges in children with non-IgE-mediated food allergies.

*Teaching Points*

- An allergy specialist dietitian can support the diagnostic process and may identify other nutrition-related issues.
- The diet history forms an important part of the diagnosis of food allergy and may direct the nature of the foods to be avoided particularly in the case of infants foods involved in delayed symptoms.
- The diet history may reveal any deficiencies which may relate to growth and development problems in infants.

**Reasons for Referral to Dietitian**

The dietitian can assist in and plays a crucial role in:

- taking an allergy-focused diet history;
- identifying any additional feeding problems either behavioural or nutritional;
- assessing nutritional intake and nutritional status;
- dietary management of any issues surrounding growth;
- practical advice on food avoidance and suitable replacements which can include:
  - foods to avoid;
  - label reading;
  - suitable substitute foods for example, egg replacers/suitable infant formulas;
  - recipe adaptation and suitable cook books;
  - internet resources;
  - support groups;
- assistance with design of food challenges;
- with young children having feeding difficulties when being weaned;
- in pregnant and lactating women following an avoidance diet for more than a few weeks, when losing weight involuntarily or when they consider stopping breastfeeding due to lack of dietary counselling and/or decrease in breast milk production.

**Case 8 (a boy with severe eczema associated with multiple food allergies)**

 

*Referral*

Jack presented at the allergy centre at age 3 months with severe eczema covering a large area of his body and face. A detailed history revealed that he had been born at full term, with a normal delivery. Jack was exclusively breastfed and his weight was between the 25th and 50th centile. His family history included asthma and hay fever in both parents. Following consultation with a paediatrician and a dietitian, Jack's mother was given information on skin care for eczema, in line with NICE guidelines [18]. Although she did not want him to have skin prick tests (SPT) at this stage, she was advised on avoidance of foods that commonly cause allergic reactions in breastfed infants, and she agreed to begin a diet excluding cow's milk and egg. In addition, advice on low-allergen weaning was provided, as Jack's mother wanted to start introducing solid foods in the coming months.

When Jack returned to the clinic at age 6 months, his mother was successfully avoiding dairy and egg. She had read advice that rice milk should be avoided for babies and toddlers, and she was drinking soya milk instead, consuming more than 500 mL each day. Jack's eczema was improving but not completely cleared, and his weight had dropped below the 25th centile. His mother also felt that he was irritable and not keen on feeding. Jack was otherwise well, with a normal physical examination and full blood count.

Jack had begun weaning at age 17 weeks, with baby rice, fruit, and vegetables. He had accidentally been given fromage frais (contains milk) by a relative and had experienced a severe immediate reaction, involving swelling of his lips and tongue, red facial flush, hives on his chest, and wheezing. Indeed, SPTs at age 6 months confirmed that Jack was sensitized to a number of foods, including milk, egg, and soya.

Given Jack's ongoing symptoms and his positive SPT to soya, the paediatrician advised his mother to begin excluding soya from her diet, while continuing to avoid egg and dairy. Jack's mother was feeling worn out by the constant effort of checking food labels and managing Jack's eczema and irritability. Consequently, she wanted to introduce a formula feed at bedtime, and an amino-acid-based formula was prescribed. Written information was provided explaining that it could be expected to taste and smell different from other formulas, and also that the change in diet may alter stool colour and consistency. If required, to aid introduction, we advised that it could be mixed with breast milk, pointing out that mixed feeds must be used immediately to avoid possible digestion by enzymes in the breast milk. Additionally, all foods, excluding dairy, egg, soya, and peanut, could be introduced into Jack's diet, one at a time. 

Jack's mother initially introduced amino-acid-based formula in a 30 : 70 mix with breast milk. The proportion of formula was gradually increased and within a few days, Jack was taking full formula feeds. At age 9 months, Jack's weight had increased to just below the 50th centile and he had a varied diet of solid foods, with breast milk and night-time feeds of amino-acid-based formula. Jack's mother was also adhering well to the exclusion diet and was taking calcium and vitamin D supplements. At age 12 months, his weight was on the 75th centile. Two days prior to the appointment, he had a reaction after eating hummus, despite the fact that his mother was eating hummus regularly and he was still receiving breast milk. The symptoms included swollen lips and a red facial flush, though there was no wheezing this time. SPTs at age 12 months confirmed that he was sensitized to sesame, among other foods. 

We advised that Jack and his mother should exclude sesame from their diets, while continuing to avoid dairy, egg, and soya. 

Jack's progress will be monitored at 6 to 12 monthly intervals, as appropriate. At each follow-up visit, SPTs and records of accidental ingestion will be used to determine whether oral challenges should take place. Deciding when to challenge can be difficult and should take into account both the clinical factors discussed here and the family's readiness, as the process can cause anxiety and emotional distress. A negative oral challenge for a given food will then enable recommendation of its introduction to Jack's diet. Clinical reactivity to milk and sesame has been confirmed by the reactions during oral exposure. The diagnosis of egg and soya allergy at this stage is based on sensitisation and improvement of symptoms after avoidance but will need to be confirmed by oral food challenges under medical supervision.

## Figures and Tables

**Table 1:** Risks for impaired growth in children with food allergy.

(i) Delayed diagnosis
(ii) Onset of disease at an early age
(iii) Multiple food allergies
(iv) Active disease
(v) Persistent (subclinical) inflammation of the gut resulting in increased requirements and/or losses and poor utilization of nutrients
(vi) Inadequate food intake due to poor appetite, regulation of gastrointestinal symptoms by modifying diet
(vii) Elimination of multiple foods from diet
(viii) Elimination of staple, nutritionally central foods from diet (milk, cereals)
(ix) Poor compliance in dietary management (unwillingness to broaden diet variety)
(x) Extreme self-restriction of foods
